# Delineating WWOX Protein Interactome by Tandem Affinity Purification-Mass Spectrometry: Identification of Top Interactors and Key Metabolic Pathways Involved

**DOI:** 10.3389/fonc.2018.00591

**Published:** 2018-12-13

**Authors:** Tabish Hussain, Jaeho Lee, Martin C. Abba, Junjie Chen, C. Marcelo Aldaz

**Affiliations:** ^1^Department of Epigenetics and Molecular Carcinogenesis, Science Park, The University of Texas MD Anderson Cancer Center, Smithville, TX, United States; ^2^Centro de Investigaciones Inmunológicas Básicas y Aplicadas, School of Medicine, Universidad de La Plata, La Plata, Argentina; ^3^Department of Experimental Radiation Oncology, The University of Texas MD Anderson Cancer Center, Houston, TX, United States

**Keywords:** WWOX, TAP-MS, interactome, WW domains, protein transport, metabolic pathways

## Abstract

It has become clear from multiple studies that WWOX (WW domain-containing oxidoreductase) operates as a “non-classical” tumor suppressor of significant relevance in cancer progression. Additionally, WWOX has been recognized for its role in a much wider array of human pathologies including metabolic conditions and central nervous system related syndromes. A myriad of putative functional roles has been attributed to WWOX mostly through the identification of various binding proteins. However, the reality is that much remains to be learned on the key relevant functions of WWOX in the normal cell. Here we employed a Tandem Affinity Purification-Mass Spectrometry (TAP-MS) approach in order to better define direct WWOX protein interactors and by extension interaction with multiprotein complexes under physiological conditions on a proteomic scale. This work led to the identification of both well-known, but more importantly novel high confidence WWOX interactors, suggesting the involvement of WWOX in specific biological and molecular processes while delineating a comprehensive portrait of WWOX protein interactome. Of particular relevance is WWOX interaction with key proteins from the endoplasmic reticulum (ER), Golgi, late endosomes, protein transport, and lysosomes networks such as SEC23IP, SCAMP3, and VOPP1. These binding partners harbor specific PPXY motifs which directly interact with the amino-terminal WW1 domain of WWOX. Pathway analysis of WWOX interactors identified a significant enrichment of metabolic pathways associated with proteins, carbohydrates, and lipids breakdown. Thus, suggesting that WWOX likely plays relevant roles in glycolysis, fatty acid degradation and other pathways that converge primarily in Acetyl-CoA generation, a fundamental molecule not only as the entry point to the tricarboxylic acid (TCA) cycle for energy production, but also as the key building block for *de novo* synthesis of lipids and amino acids. Our results provide a significant lead on subsets of protein partners and enzymatic complexes with which full-length WWOX protein interacts with in order to carry out its metabolic and other biological functions while also becoming a valuable resource for further mechanistic studies.

## Introduction

The *WWOX (*WW *domain-containing* OX*idoreductase)* gene, spans 1.1 Mb, and contains common chromosomal fragile site FRA16D at ch16q23.1-23.2 (1, 2). It encodes a 414-amino acid, 46-kDa protein composed of two WW domains in tandem (designated WW1 and WW2) located N-terminal to the short-chain dehydrogenase/reductase (SDR) domain (1). *WWOX* was originally discovered by our laboratory and described by us and others as a putative tumor suppressor protein mostly associated with tumor progression, and therapy resistance in multiple cancer types (3–6). It has become clear by experimental evidence from multiple studies and human data that WWOX operates as a “non-classical” tumor suppressor [Reviewed in (5, 7)], likely of more relevance for affecting tumor progression rather than cancer initiation. Importantly, over the years *WWOX* has become recognized for its role in a much wider array of human pathologies including metabolic conditions and central nervous system (CNS) related syndromes (5, 8–10). WWOX is ubiquitously expressed in various tissues and cell types (11) and has been suggested to play roles in multiple cellular processes including but not limited to: apoptosis (12, 13); regulating the availability of various transcription factors, cofactors, and signaling molecules (14–19), cell adhesion (20, 21), metabolic functions (5, 10, 22, 23), and maintenance of genomic stability (6, 24). In most of the described cellular processes, WWOX was proposed to exert its regulatory/homeostatic roles *via* direct protein-protein interactions. Therefore, the apparent versatile nature of WWOX can be attributed in part to its ability to interact with different proteins in multiple cellular pathways. However, it is noteworthy that the significance of most of the reported interactions to the most relevant biological functions of WWOX in the normal cell still remains to be settled.

In early studies, we defined the characteristics of the prime candidate domains within the WWOX protein structure that drive protein-protein interactions, i.e., the WW domains. WW domains are small protein modules named for their unique structure: two conserved tryptophan (W) residues spaced ~20–22 amino acids apart (25). These domains are typically 35–40 amino acids in length and fold into a three-stranded, antiparallel β-sheet with two ligand-binding grooves (25, 26). WW domains were originally classified into four classes depending on their binding affinity to a diverse set of proline-rich ligand consensus motifs: Group I binding preferentially PPXY (27), Group II binding PPLP (28) (where P is proline, Y is tyrosine, L is leucine, and *X* is any amino acid), Group III binding poly-proline sequences flanked by Arg or Lys (29), and Group IV binding phospho-Ser-Pro or phospho-Thr-Pro (30). We determined that WWOX WW1 domain, conformed by tryptophan (W) residues at positions 22 and 44, belongs to Group I due to its predilection for binding proteins harboring PPXY motifs (31). WWOX WW2 domain is not a classical WW domain due to the replacement of the second signature tryptophan by a tyrosine at position 85 and no binding motif was identified (31). More recently, it was further confirmed that the WW2 domain does not bind to any consensus proline-rich motif, but does augment the ability of WW1 to do so (32).

The vast majority of studies utilized low throughput protein-protein interaction assays to identify potential WWOX interacting partners, such as affinity capture-western, reconstituted complex, and yeast two-hybrid systems. Only two high throughput scale studies were reported where GST-fusion WWOX WW1 domain protein constructs were used as bait and its interacting partners were precipitated through GST-pulldown approaches followed by identification using mass spectrometry (MS) (33, 34). The report of Ingham et al. was done *in-vitro* on cell extracts (33) while the study of Abu-Odeh et al. was done after ectopically expressing a GST-WW1 domain fusion construct in HEK293 cells followed by GST-pulldown and MS (34). Although important in their own way, some limitations are intrinsic to the mentioned approaches and this include: (i) the use of only a very small portion of the WWOX protein (i.e., WW1 domain); (ii) the large size of the fusion GST tag (221 amino acids, 22-kDa) with potential non-physiological folding of the ectopically expressed fusion protein (i.e., WW1 domain + GST tag); and (iii) lack of the functional enzymatic SDR domain known to significantly affect intracellular localization (5, 35).

The key question regarding the myriad of reported WWOX interacting proteins identified both by low and high throughput approaches is how many are truly relevant and really reflect the most important biological functions of WWOX in the normal cell. It is important to stress that WWOX is also an enzyme predicted to carry out NAD(H) or NADP(H)-dependent dehydrogenase reactions with yet to be identified substrate/s, and undoubtedly this predicted metabolic role of WWOX is likely to be of much relevance to ultimately answer the aforementioned question.

The development of high-throughput Tandem Affinity Purification-Mass Spectrometry (TAP-MS) methods has revolutionized proteomics research (36, 37). As an unbiased approach, TAP-MS technology is ideal for the identification of not only the individual bait interacting proteins but also larger protein complexes “associated” with specific bait-protein partner pairs. In this article, we report using a TAP-MS approach with full-length WWOX protein as bait in order to identify WWOX-protein/complex interactions under physiological conditions on a proteomic scale. This work led to the identification of both well-known and novel WWOX interacting partners, suggesting the involvement of WWOX in multiple specific biological and molecular processes while delineating a comprehensive portrait of WWOX's protein interaction network (i.e., WWOX interactome) and becoming a valuable resource for further mechanistic studies on the key WWOX biological functions.

## Materials and Methods

### Vector Construct, Cell Culture, Transfection and Clone Selection for TAP-MS

HEK293T cells stably expressing SBP-S-FLAG (SFB) triple-tagged WWOX were generated as described previously (38). Briefly, the cDNA WWOX full reading frame construct (1) was subcloned into a pDONOR201 vector using Gateway Technology (Invitrogen, Carlsbad, CA, United States) as the entry clone. Next, a lentiviral-gateway-compatible destination vector was used to recombine WWOX entry clone for expression of a C-terminal triple (S tag-Flag tag-SBP tag, i.e., SFB) tagged fusion protein. All constructs were sequence verified. HEK293T cell line was obtained from the American Type Culture Collection (ATCC) and maintained in Dulbecco modified essential medium (DMEM) supplemented with 10% fetal bovine serum at 37°C in 5% CO_2_. Using polyethylenimines the SFB-tagged WWOX encoding vector construct was transfected into HEK293T cells. Cells were selected with puromycin, 12 single clones were picked and examined for WWOX expression by Western blotting using anti-FLAG antibody (MilliporeSigma, Burlington, MA, United States).

### Tandem Affinity Purification of WWOX Interacting Protein/Complexes and MS Analysis

The TAP-MS procedure has been previously described in detail (38–41). Briefly, HEK293T cells stably expressing SFB-tagged WWOX were lysed with NETN lysis buffer. For the first affinity purification step, crude lysate was subjected to centrifugation and the supernatant was incubated with streptavidin-conjugated beads (GE Healthcare, Pittsburg, PA, United States). The beads were washed three times with NETN buffer, and the bound proteins were eluted with NETN buffer containing 2 mg/ml biotin (MilliporeSigma, Burlington, MA, United States). For the second purification step, eluate obtained in step 1 was incubated with S protein beads (Novagen, Kenilworth, NJ, United States). The beads were washed again with NETN buffer thrice and subjected to SDS-PAGE. The protein band containing the entire sample was excised, chopped, and gel pieces were subjected to in-gel trypsin digestion followed by drying. Dried sample was reconstituted in 5 μl of HPLC solvent A (2.5% acetonitrile, 0.1% formic acid). A nano-scale reverse-phase HPLC capillary column was created by packing 5 μm C18 spherical silica beads into a fused silica capillary (100 μm inner diameter x ~20 cm length) with a flame-drawn tip. After equilibrating the column, the sample was loaded *via* a Famos autosampler (LC Packings, San Francisco, CA, United States) onto the column. A gradient was created and peptides were eluted with increasing concentrations of solvent B (97.5% acetonitrile, 0.1% formic acid). As the peptides eluted, they were subjected to electrospray ionization and then entered into an LTQ Velos ion trap mass spectrometer (Thermo Fisher, San Jose, CA, United States).

Peptide sequences (protein identity) were determined by matching the acquired fragmentation pattern with protein databases by the software program, SEQUEST (ver. 28) (Thermo Fisher) and spectral counts were generated. To evaluate potential protein-protein interactions, assign probabilistic scores to individual interactions and eliminate non-specific interactions, the Minkowski distance-based unified probabilistic scoring environment (MUSE) statistical model was applied. Next, to remove background noise contaminants the data was analyzed using the CRAPome database (https://www.crapome.org). The detailed procedure for CRAPome proteomic data analyses is described elsewhere (42). Identification of WWOX WW1 domain putative binding motifs in interacting partners was done using the ExPASy-PROSITE web resource that offers tools for protein sequence analysis and motif detection (43). Biological pathway analysis was done using the Innate Database (http://www.innatedb.com)(44).

### Co-immunoprecipitation

Full open reading frame, sequenced verified Myc-DDK-VOPP1 (Catalog # RC221464), Myc-DDK-SCAMP3 (Catalog # RC201633), and Myc-DDK-SEC23IP (Catalog # RC209056) expression construct plasmids were purchased from OriGene (Rockville, MD, United States). Amino-terminal GFP-WWOX plasmid construction was previously described (3). Anti-Myc and anti-GFP antibodies were purchased from Cell Signaling (Danvers, MA, United States) and Invitrogen (Waltham, MA, United States), respectively. The anti-WWOX rabbit polyclonal monospecific primary antibody was developed in our laboratory (31). For co-immunoprecipitation, HEK293T cells were co-transfected with either 1 μg of Myc-DDK-VOPP1, Myc-DDK-SCAMP3, or Myc-DDK-SEC23IP along with 1 μg of GFP-WWOX plasmid. After 36 h cells were lysed with lysis buffer (50 mM Hepes, pH 7.4, 150 mM NaCl, 1.5 mM MgCl_2_, 1 mM EGTA, 1% Triton X-100, 50 mM NaF, 2 mM Na_3_VO_4_, and 10% glycerol) containing 1 × complete protease inhibitor mixture (Roche, Indianapolis, IN). After centrifugation (12,000 g at 4°C for 10 min), total cell lysate supernatant fractions were collected and ~600 μg of total protein was incubated with respective antibodies at a 1:200 dilution for 2 h followed by incubation with Protein A/G PLUS-Agarose (Santa Cruz, Dallas, TX, United States) for 16 h at 4°C on a rotary shaker. Corresponding IgG (MilliporeSigma, Burlington, MA, United States) was used as a negative control. Antibody-bound beads were washed three times and bound protein complexes precipated. A 3.3% of the total cell lysate was used as input and 50% of immuno-precipitated protein sample was used for western blotting using 1:2,000 dilution of anti-Myc, anti-GFP, or anti WWOX antibodies.

### GST Pull-Down Assay

GST-fusion protein constructs: (i) wild-type WW domains, i.e., WW1 + WW2 (GST-WW1-2); (ii) mutant WW1 domain (GST-Mut-WW1), i.e., WW1 W44F/P47A + WW2 WT; (iii) mutant WW2 domain (GST-Mut-WW2), i.e., WW1 WT + WW2 Y85A/P88A; (iv) WW1 WT (GST-WW1); (v) WW2 WT (GST-WW2); (vi) and full length wild-type WWOX (GST-WWOX) were constructed and purified as previously described (31). GST-fusion proteins were expressed in *Escherichia coli* strain BL21 and purified as described by the manufacturer (MilliporeSigma, Burlington, MA, United States). Cleared bacterial lysates from 100 ml cultures were made by sonication in PBS containing 1x complete protease inhibitor mixture (Roche, Indianapolis, IN, United States). GST-fusion proteins were purified using Glutathione Sepharose 4B (MilliporeSigma, Burlington, MA, United States). NP-40 to a final concentration of 0.1%. was added to the bacterial lysates. Cleared total cell lysates from HEK293T cells were prepared using lysis buffer (50 mM Tris–Cl, pH 7.5, 150 mM NaCl, 1 mM EDTA, 1% Triton X-100, and 10% glycerol). GST pull-down assays were performed by addition of 10 μg of purified GST-fusion protein and 20 μl of Glutathione Sepharose 4B (50% slurry) to 600 μg of total cell lysate from HEK293T cells. After incubation, overnight at 4°C with mixing, the beads were washed three times with cold lysis buffer and once with cold PBS. Twenty micrograms of bound proteins were separated by SDS/PAGE and analyzed with Western blotting using 1:2,000 anti-Myc antibody.

### Statistical Methods

The comparison of interaction scores between WWOX binding proteins with- and without-motif was done using statistical software GraphPad PRISM 7 and the Student's two-tailed unpaired *t*-test was used for comparisons. Statistical values of the enriched pathways were obtained through analysis with InnateDB. *P* < 0.05 were considered significant.

## Results

### TAP-MS Based Proteomic Profiling of WWOX Interactome

**Figure 1:**
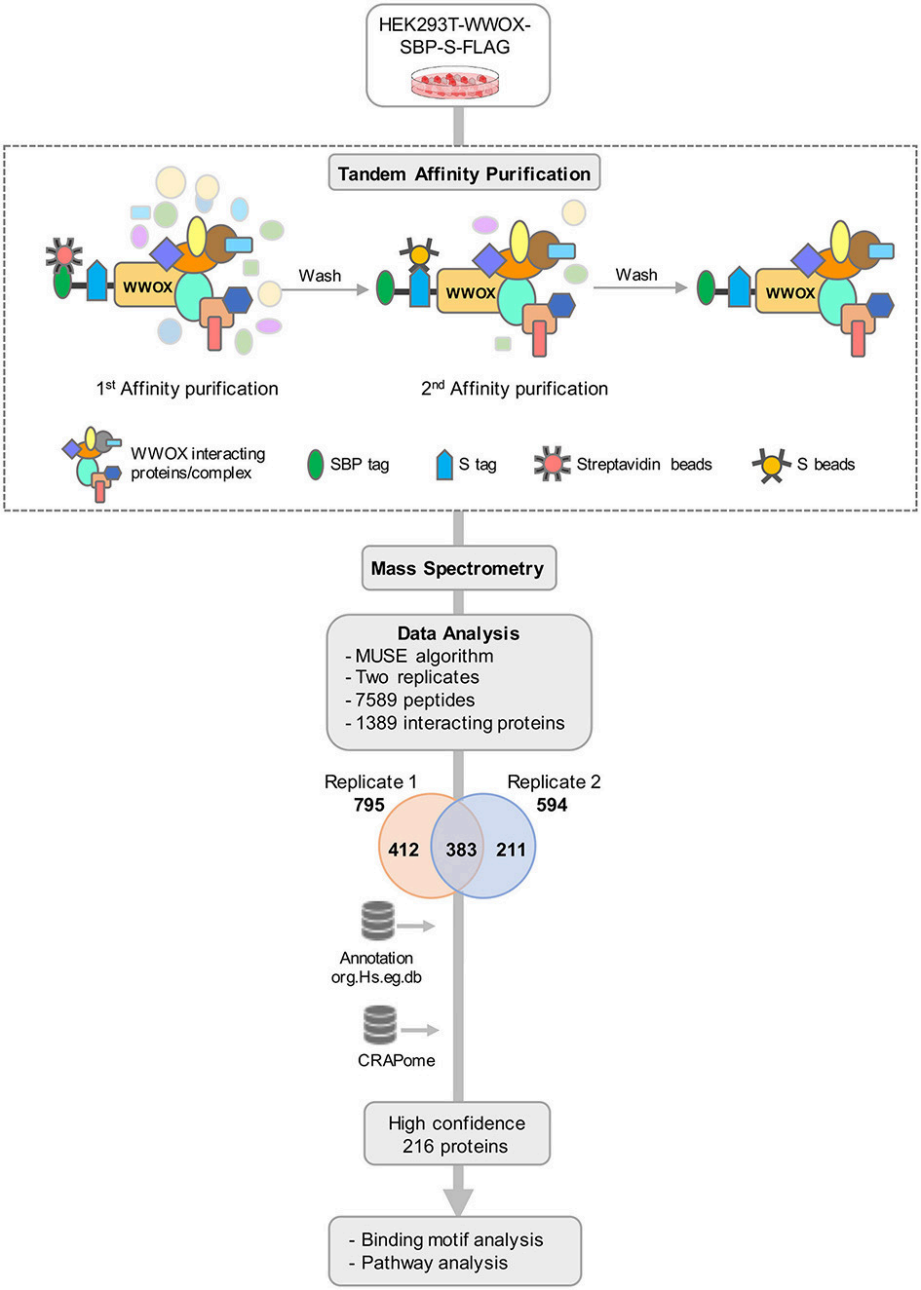
TAP-MS based Proteomic Profiling of WWOX Interactome. Schematic diagram showing the major steps involved in the TAP-MS procedure and data analysis. HEK293T cells stably expressing WWOX with C-terminal SFB-tag (Streptavidin binding protein (SBP)-tag, S-tag, and Flag-tag) were generated by stable transfection and puromycin selection. Following the described tandem affinity purification steps, purified protein complexes were identified by mass spectrometry analysis (LC-MS/MS), and final interacting proteins were identified after proper annotation and MUSE algorithm-based analysis to define interaction scores. Data annotation was done using Bioconductor. To remove the non-specific bindings or contaminants data was analyzed using CRAPome database to obtain a list of high confidence 216 binding partners. Finally, data were subjected to motif and pathway analysis.

To obtain a comprehensive view of the human WWOX protein interactome, we performed TAP-MS analyses as illustrated in the stepwise summary shown in Figure 1. We used HEK293T cells stably expressing SBP-S-FLAG (SFB) triple-tagged WWOX as previously described by expression construct transfections followed by puromycin selection (38). Twelve cell clones were isolated and examined for expression levels of tagged WWOX by immunoblotting and selected those with similar expression level as the endogenous WWOX protein to proceed with the TAP steps. Total cell lysates from cells stably expressing SFB-tagged WWOX were subjected to two rounds of affinity purifications using streptavidin beads and S-beads, the final eluate was analyzed by LC-MS/MS. We identified a total of 7,589 peptides from two replicates, corresponding to a total of 795 proteins in replicate-1 and 594 proteins in replicate-2 (Supplementary File 1). A total of 1,006 unique interacting proteins were identified from the sum of both replicates. Next, to increase the probability of identifying true binding partners and protein complexes we focused and annotated a total of 383 proteins that were common between replicates 1 and 2 for further analysis (Figure 1).

In order to refine the list of WWOX interactors, we further analyzed our dataset using the Contaminant Repository for Affinity Purification database (https://www.crapome.org). CRAPome is a web-accessible resource that stores annotated negative controls generated using 411 affinity purification based MS experiments performed by the proteomics research community, and enables analyzing affinity purification based MS data for ultimately providing a CRAPome frequency score as described in detail by Mellacheruvu et al. (42). Briefly, a lower CRAPome frequency score indicates a higher specificity of the prey protein. From the identified 383 proteins, we narrowed down the list to 253 proteins with a CRAPome frequency < 0.2. The data was further shortlisted based on the MUSE algorithm generated interaction scores (40) thus generating a final list of 216 WWOX prey proteins provided in Supplementary File 2.

### High Confidence WWOX Protein Interactors

**Figure 2:**
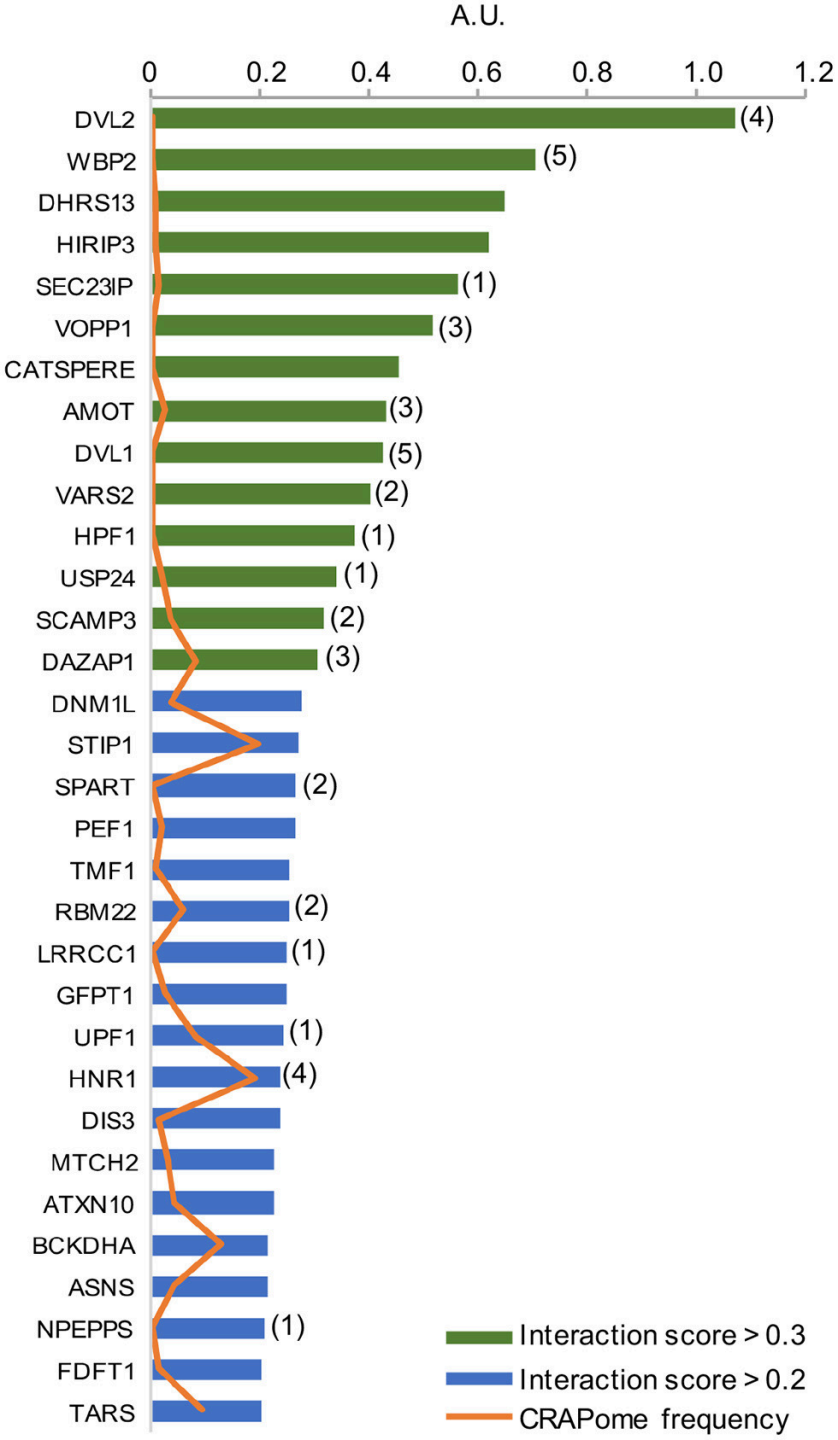
High confidence WWOX protein interactors. Graph showing candidate WWOX interacting partners with interaction score (IS) > 0.2. Bars in green represent the set of 14 proteins with an extremely high likelihood of being direct physical WWOX binding proteins with IS > 0.3. Bars in blue represent good candidate WWOX direct binding proteins with IS < 0.3 and > 0.2. Numbers in parenthesis next to bars represent the number of potential WWOX binding motifs for each respective interacting protein. The orange line graph represents CRAPome frequency for each WWOX binding protein.

As per analysis with the MUSE algorithm (40), we identified 14 proteins with TAP-MS interaction scores (IS) > 0.3. These targets have a very high likelihood of being direct WWOX physical binding partners (Table 1 and Figure 2). An additional set of 18 good candidate WWOX binding proteins with IS < 0.3 and > 0.2 are also shown in Table 1 and Figure 2. These proteins have varied functions including signal transduction molecules, enzymes, nucleic acid binders, or transporters and exhibit diverse sub-cellular localizations including, cellular membrane, cytoplasm, nucleus, Golgi apparatus, endoplasmic reticulum, mitochondria, and cell junctions. Not unexpectedly, among the highest confidence group, we find previously described WWOX binding proteins such as DVL2, DVL1, and AMOT (17, 34), with DVL2 displaying by far the strongest interaction score with WWOX. Other previously described WWOX interactors can be found in this high confidence dataset such as WBP2 (45), and as will be described below some of these top candidates were identified as well by a previous high throughput study (34). However, the vast majority of the 216 proteins we identified using TAP-MS shown in Supplementary File 2 have not been previously reported as WWOX interactors. It is worth noting that among the 14 highest confidence protein interactors (IS > 0.3) 80% (i.e., 11 of 14 proteins) contain candidate WWOX WW1 domain binding motifs (Table 1, green bars in the graph- Figure 2). This observation reinforces the notion that this dataset can help us identify novel and relevant direct WWOX binding proteins *via* WW domains. On the other hand, among the list of top interactors with IS > 0.2, 15 of 32 proteins do not display canonical WW domain binding motifs suggesting that physical interactions may occur *via* other WWOX protein regions or some of these prey proteins could be members of larger protein complexes.

**Table 1:** Candidate WWOX interacting proteins with highest confidence interaction scores.

**Symbol**	**Protein name**	**Subcellular localization**	**Interaction score**	**Candidate binding motifs**
DVL2	Disheveled 2	Cytosol/Nucleus/Plasma membrane	1.07	^133^PPSF^136^, ^565^PPPY^568^, ^78^LPCF^81^, ^548^LPTF^551^
WBP2	WW domain-binding protein 2	Cytoplasm/Nucleus	0.70	^167^PPGY^170^, ^176^PPEF^179^, ^197^PPPY^200^, ^249^PPPY^252^, ^250^PPYY^253^
DHRS13	Dehydrogenase/ reductase SDR family member 13	Cytoplasm/Secreted	0.65	–
HIRIP3	HIRA-interacting protein 3	Nucleus	0.62	–
SEC23IP	SEC23-interacting protein	Endoplasmic reticulum	0.56	^164^PPSY^167^
VOPP1	Vesicular, Overexpressed in Cancer, Pro-survival Protein 1	Cytoplasmic vesicle membrane	0.51	^116^PPYY^119^, ^154^PPAY^157^, ^162^PPPY^165^
CATSPERE	Catsper Channel Auxiliary Subunit Epsilon	Plasma membrane	0.45	–
AMOT	Angiomotin	Cell junction/Tight junction	0.42	^239^PPEY^242^, ^284^PPEY^287^, ^106^LPTY^109^
DVL1	Disheveled 1	Cytosol/Plasma membrane/Cytoplasmic vesicle	0.42	^117^PPSF^120^, ^546^PPCF^549^, ^550^PPAY^553^, ^70^LPCF^73^, ^374^LPRY^377^
VARS2	Valyl-tRNA synthetase	Mitochondrion	0.39	^101^PPAY^104^, ^22^LPRF^25^
HPF1	Histone PARylation Factor 1	Nucleus	0.37	^112^PPEF^115^
USP24	Ubiquitin carboxyl-terminal hydrolase 24	Nucleus	0.33	^98^PPAY^101^
SCAMP3	Secretory Carrier Membrane Protein 3	Endosome/Exosome/Secreted	0.31	^50^PPAY^53^, ^138^LPSF^141^
DAZAP1	DAZ Associated Protein 1	Nucleus	0.30	^259^PPPF^262^, ^287^PPQF^290^, ^370^PPSY^373^
DNM1L	Dynamin 1 Like protein	Cytosol/Peroxisome/Mitochondrion/Golgi apparatus/	0.27	–
STIP1	Stress Induced Phosphoprotein 1	Nucleus	0.26	–
SPART	Spartin	Cytoplasm	0.26	^171^PPAY^174^, ^265^PPGF^268^
PEF1	Peflin	Endoplasmic reticulum	0.26	–
TMF1	TATA element modulatory factor 1	Nucleus/ Golgi apparatus	0.25	–
RBM22	RNA Binding Motif Protein 22	Nucleus	0.25	^376^PPGF^379^, ^390^PPPF^393^
LRRCC1	Leucine Rich Repeat and Coiled-Coil Centrosomal Protein 1	Cytoskeleton	0.24	^180^LPGY^183^
GFPT1	Glutamine-fructose-6-phosphate aminotransferase 1	Cytosol/Secreted	0.24	–
UPF1	Regulator of non-sense transcripts 1	Cytoplasm	0.24	^1005^PPGY^1008^
HNRL1	Heterogeneous nuclear ribonucleoprotein U-like protein 1	Nucleus	0.23	^714^PPSY^717^, ^781^PPAY^784^, ^834^PPYY^837^, ^394^LPGF^397^
DIS3	Ribosomal RNA-Processing Protein 44	Nucleus	0.23	–
MTCH2	Mitochondrial Carrier 2	Mitochondrion	0.22	–
ATXN10	Ataxin 10	Cytosol/ Secreted/ Plasma membrane	0.22	–
BCKDHA	Branched Chain Keto Acid Dehydrogenase E1, Alpha Polypeptide	Mitochondrion	0.21	–
ASNS	Asparagine synthetase	Cytosol	0.21	–
NPEPPS	Puromycin-Sensitive Aminopeptidase	Cytosol/ Nucleus	0.20	^289^LPFY^292^
FDFT1	Farnesyl-Diphosphate Farnesyltransferase 1	Endoplasmic reticulum	0.20	–
TARS	Threonyl-tRNA Synthetase	Cytoplasm	0.20	–

### WWOX Physical Interaction With Members of Protein Complexes Related to Protein Trafficking From ERES to Golgi and From Late Endosomes to Lysosomes

**Figure 3:**
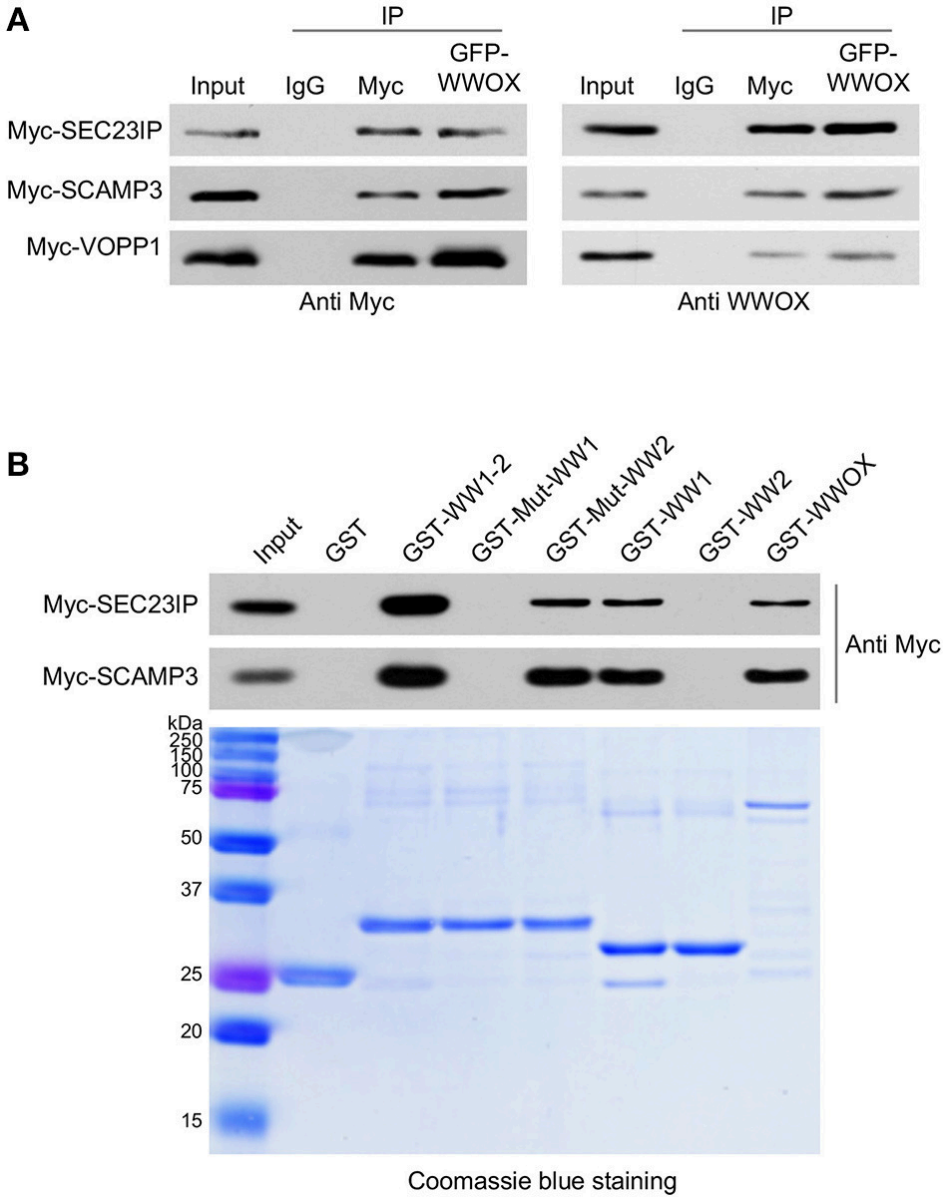
**(A)** Direct physical interaction between WWOX and SEC23IP, SCAMP3, and VOPP1 proteins. Reciprocal co-immunoprecipitation of GFP-WWOX and Myc-SEC23IP, Myc-SCAMP3 or Myc-VOPP1 in HEK293T cells. Cells were co-transfected with either 1 μg of Myc-DDK-VOPP1, Myc-DDK-SCAMP3, or Myc-DDK-SEC23IP along with 1 μg of GFP-WWOX plasmid. After 36 h transfected cells were lysed and were immunoprecipitated with either rabbit IgG (control), anti-Myc, or anti-GFP antibodies. The immunoprecipitates were immunoblotted with anti-Myc or anti-WWOX antibodies as indicated. **(B)** Western blot and corresponding Coomassie blue staining of GST pull-down assay. HEK293T cells were transfected with either Myc-SEC23IP or Myc-SCAMP3 expression vectors. Cell lysates were subjected to pulldowns with the indicated GST fusion proteins: either wild-type WW domains, i.e., WW1 + WW2 (GST-WW1-2); mutant WW1 domain (GST-Mut-WW1), i.e., WW1 W44F/P47A + WW2 WT; mutant WW2 domain (GST-Mut-WW2), i.e., WW1 WT + WW2 Y85A/P88A; WW1 WT (GST-WW1), WW2 WT (GST-WW2), full length wild-type WWOX (GST-WWOX), or GST alone. Bound protein was detected by anti-Myc immunoblot.

We have previously shown that WWOX is predominantly a cytoplasmic protein localizing to the perinuclear compartment significantly overlapping with the Golgi region (3, 31). Interestingly, among the most likely direct WWOX interactors identified by TAP-MS, at least three proteins are related to the endoplasmic reticulum (ER), Golgi, late endosomes, protein transport, and lysosomes networks: SEC23IP (SEC23-interacting protein), SCAMP3 (Secretory Carrier Membrane Protein 3), and VOPP1 (Vesicular, Overexpressed in Cancer, Pro-survival Protein 1). All these WWOX interacting proteins have an IS > 0.3 and based on PROSITE analyses, all of them contain canonical WW domain binding motifs (Table 1). We validated the direct physical interaction of WWOX with these three proteins by means of co-immunoprecipitation (co-IP) in human cells. Myc-tagged expression constructs for each protein were co-transfected with a GFP-tagged WWOX expression vector in HEK-293T cells. Cell lysates were immunoprecipitated with anti-Myc or anti-GFP antibodies and immunostained with anti-Myc or anti-WWOX antibodies. Indeed, co-IP of WWOX with SEC23IP, SCAMP3, and VOPP1 proteins demonstrated a strong physical interaction between WWOX and each of these proteins. Furthermore, reciprocal co-IP with all of the partners for each interaction was observed (Figure 3A). Very recently, Bonin et al. (46) have also identified VOPP1 as a WWOX interactor in human cells by means of yeast-two hybrids and demonstrated that the WW1 region is the interacting domain mostly with the ^162^PPPY^165^ motif of VOPP1.

In order to determine whether the observed interaction of WWOX with SEC23IP and SCAMP3 is also dependent on WWOX WW1 domain, GST-pull down experiments were performed. We observed that SEC23IP and SCAMP3 from HEK-293T whole cell lysates readily bind to GST-fusion constructs of full-length wild-type WWOX (GST-WWOX), wild-type GST-WW1-2, and wild-type GST-WW1 (Figure 3B). On the other hand, no binding was observed to GST-Mut-WW1 (WW1 W44F/P47A + WW2 WT) or wild-type GST-WW2. Thus, the interaction with SEC23IP and SCAMP3 is lost when key functional residues of the WW1 domain are mutated, while the mutation in the WW2 domain (GST-Mut-WW2) does not affect the overall binding (Figure 3B). In summary, these results demonstrate that SEC23IP and SCAMP3 are indeed novel direct interacting partners of WWOX *via* WW1 domain.

### WWOX WW1 Binding Motifs in TAP-MS Dataset

**Figure 4:**
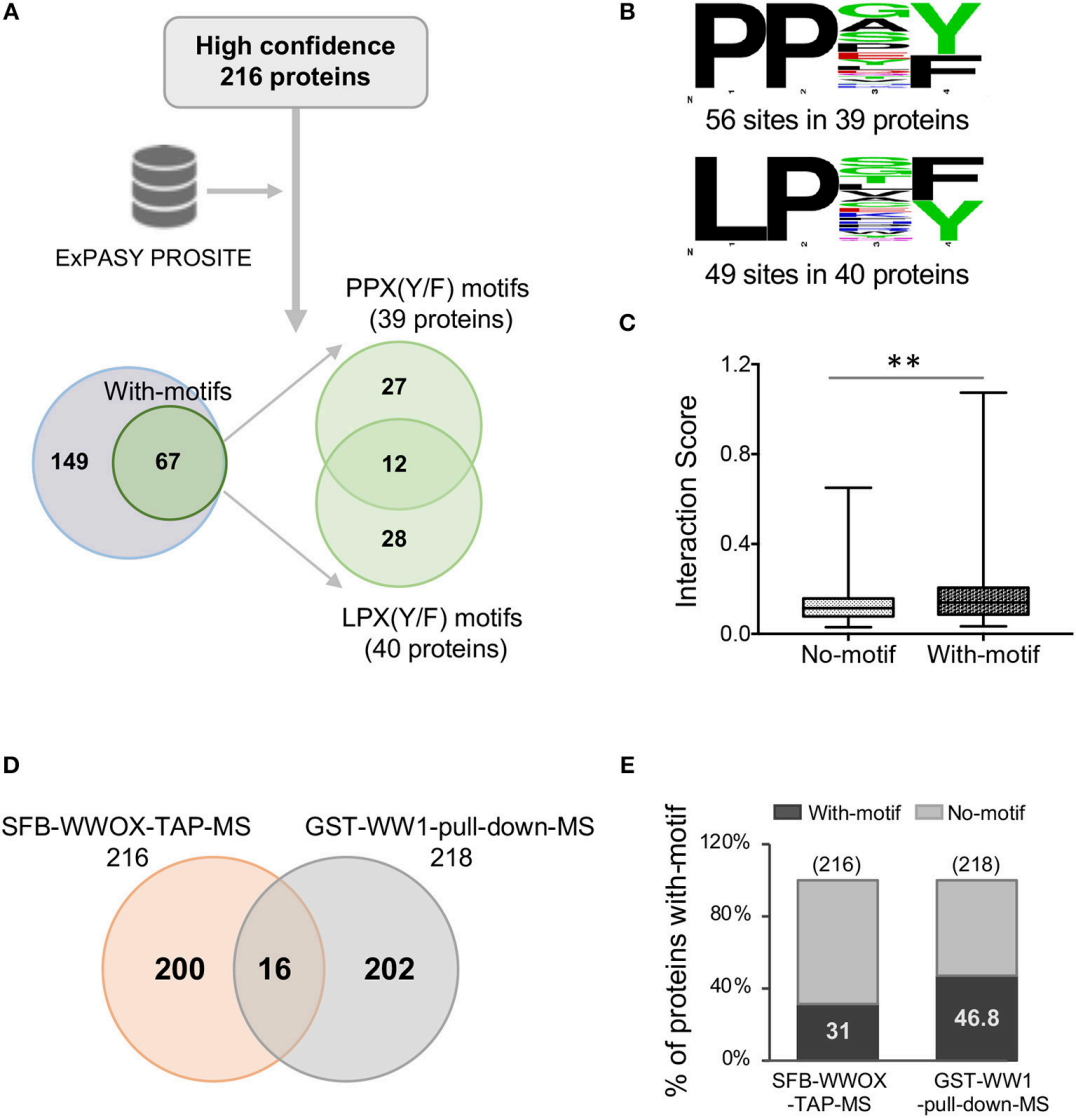
WW domain binding motifs in WWOX interacting proteins. **(A)** ExPASY PROSITE analysis was performed on the list of 216 WWOX interactors (Supplementary File 2). Candidate WWOX WW1 domain binding motifs were identified in 31% (67 out of 216) proteins. Out of 67 proteins with putative WW1 binding motifs, 12 candidate WWOX binding interactors displayed both PPX(Y/F) and LPX(Y/F) motifs. **(B)** Amino acid sequence of candidate WWOX WW1 domain binding motifs. The probability of the residue at each position is proportional to the size of the letters. The image was generated using the Weblogo 3.4 program. Thirty-nine proteins displayed PPX(Y/F) motifs at 56 sites and 40 proteins displayed LPX(Y/F) motif at 49 sites. **(C)** Box and whisker plot showing the comparison of median interaction scores between proteins with- and without-motifs. Proteins with-motifs display higher interaction scores (mean interaction score = 0.1875) vs. proteins with No-motifs (mean interaction score = 0.1287). Statistical analysis was done using two-tailed unpaired Student's *t*-test, ^**^p < 0.001. **(D)** Comparison of SFB-WWOX-TAP-MS data with GST-WW1-pull-down-MS data from Abu-Odeh (34). Only 16 common WWOX interactors were identified overlapping both datasets. **(E)** Bar graph showing a comparison of the percentage of proteins with WWOX binding motifs between the aforementioned datasets. Thirty-one percent of proteins from the SFB-WWOX-TAP-MS dataset (this study) displayed candidate WW1 binding motifs vs. 46.8% of proteins in the GST-WW1-pull-down-MS dataset (34).

A previous study by Abu-Odeh et al. (34) employed an MS-based screen utilizing GST-WWOX WW1 domain constructs as bait which confirmed the predilection of the WW1 domain to bind proteins with PPXY motifs. However, they also observed that many of WWOX WW1 interacting proteins did not contain PPXY motifs but exhibited instead PPXF, LPXY, or LPXF motifs suggesting that the WW1 domain of WWOX binds also non-canonical class 1 WW proline-containing motifs. We examined the prevalence of potential WW domain binding motifs as defined in the aforementioned study among the 216 proteins identified in our TAP-MS experiment. Analysis of these candidate WWOX interacting partners using PROSITE showed that 31% (67 out of 216) proteins contained canonical WW1 domain putative PY binding motifs as well as other proline-containing motifs

(Figure 4A), as previously described (34). Of a total of 67 proteins, 12 candidate WWOX binding partners displayed both PPX(Y/F) and LPX(Y/F) motifs (Figure 4A). Thirty-nine proteins displayed PPX(Y/F) motifs at 56 sites and 40 proteins displayed LPX(Y/F) motifs at 49 sites (Figure 4B and Supplementary File 3). Notably, 69% (149 out of 216) WWOX candidate binding proteins were devoid of any putative WW1 domain binding motifs. We compared the interaction scores of proteins with- and without-motifs. Proteins with-motifs displayed a significantly higher interaction score (mean interaction score = 0.1875, *p* < 0.001) as compared to proteins with no-motifs (mean interaction score = 0.1287, Figure 4C), thus suggesting as expected a higher affinity for direct physical WWOX binding.

We also compared our full-length WWOX TAP-MS dataset with the previous GST-WW1 pull-down report (34). In the Abu-Odeh et al. study 218 WWOX interactors were identified as reported in BioGRID (https://thebiogrid.org/) (34). Surprisingly, we identified only 16 common interactors (Table 2 and Figure 4D) comparing both studies. Eight of these 16 proteins were also found among our high-to-good confidence candidate WWOX binding proteins with interaction scores >0.2. Ten of these 16 proteins harbored candidate proline-containing binding motifs (Table 2). We also compared the prevalence of candidate WWOX WW1 binding motifs among the putative interactors between the two datasets (Figure 4E).

**Table 2:** Common WWOX interactors identified in TAP-MS (this study) and Abu-Odeh et al. (34) datasets.

**Symbol**	**Protein name**	**Interaction score**	**Binding motifs**
DVL2	Disheveled 2	1.07	Yes
WBP2	WW domain-binding protein 2	0.70	Yes
SEC23IP	SEC23-interacting protein	0.56	Yes
DVL1	Disheveled 1	0.42	Yes
AMOT	Angiomotin	0.42	Yes
DAZAP1	DAZ Associated Protein 1	0.30	Yes
SPART/ SPG20	Spartin	0.26	Yes
HNRL1/ HNRNPUL1	Heterogeneous nuclear Ribonucleoprotein U-like protein 1	0.23	Yes
FARSA	Phenylalanine-tRNA ligase alpha subunit	0.16	–
ATP2A1	Endoplasmic reticulum calcium ATPase 1	0.15	–
HSD17B10	3-hydroxyacyl-CoA dehydrogenase type-2	0.13	–
RAPGEF2	Rap guanine nucleotide exchange factor 2	0.12	Yes
TARDBP	TAR DNA-binding protein 43	0.09	–
ATAD3A	ATPase family AAA domain-containing protein 3A	0.08	–
HADHA	Trifunctional enzyme subunit alpha, mitochondrial	0.06	–
CYFIP1	Cytoplasmic FMR1-interacting protein 1	0.04	Yes

### Metabolic Pathways Associated With WWOX Protein Interactome

**Figure 5:**
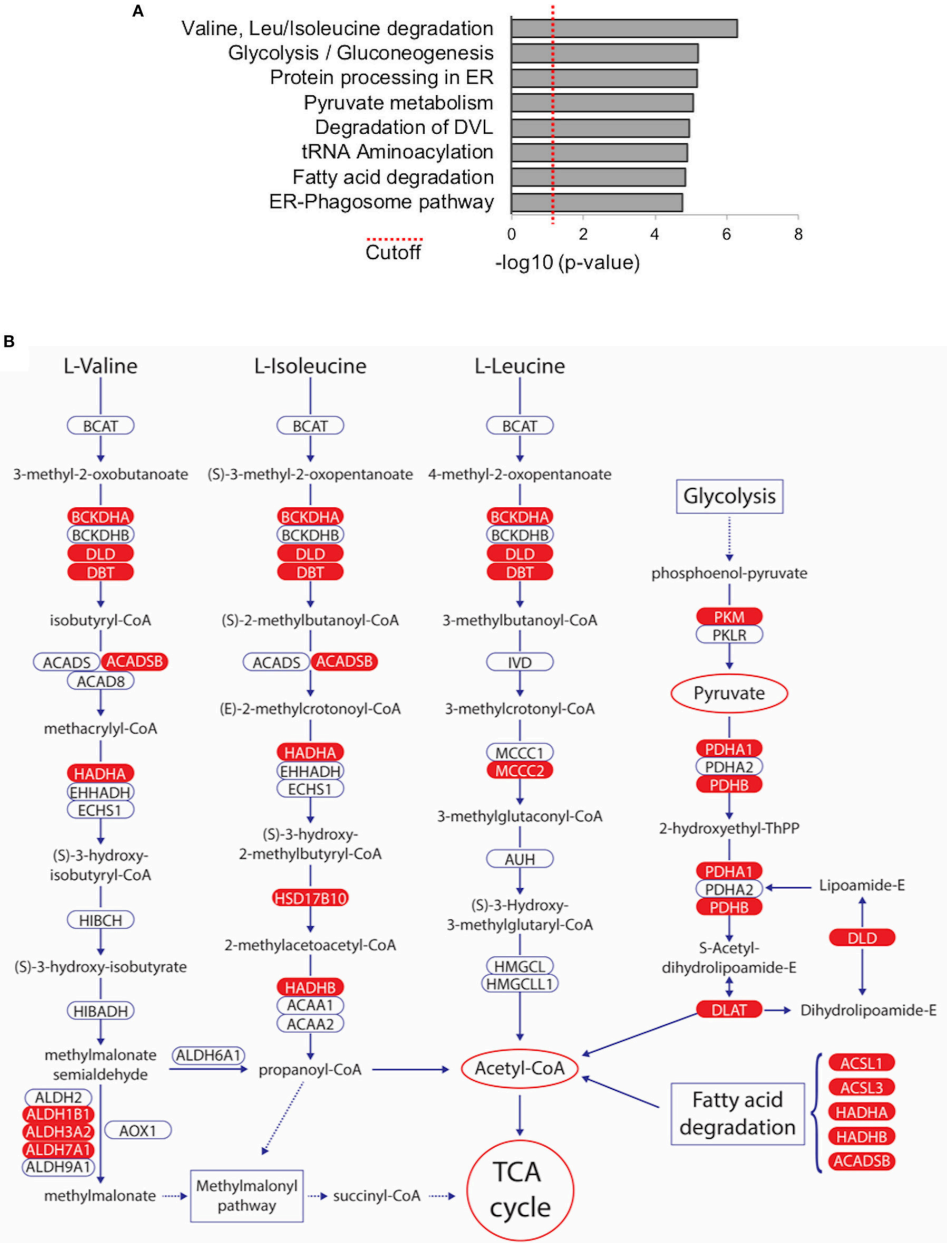
Metabolic Pathways significantly associated with WWOX protein interactome. **(A)** Bar graph represents biological pathways that are significantly over-represented within the list of 216 WWOX interactors. See Supplementary File 4 for detail of interactors and *p*-values of pathway shown **(B)** Among the most significantly associated pathways we identified key metabolic pathways including: “Valine, Leucine, and Isoleucine degradation” (KEGG pathway hsa00280), “Glycolysis/Gluconeogenesis” (KEGG pathway hsa00010), “Pyruvate metabolism” (KEGG pathway hsa00620), and “Fatty acid degradation” (KEGG pathway hsa00071). All pathways converge to Acetyl-CoA generation the key molecule that delivers its acetyl group for oxidation and energy production to the tricarboxylic acid (TCA) cycle. WWOX interactors identified in our TAP-MS dataset are highlighted in red.

In order to investigate if any biological pathways are significantly over-represented within the list of 216 TAP-MS identified WWOX binding proteins, we used the Innate Database (http://www.innatedb.com) (44). Among the most significantly associated pathways we identified key metabolic pathways including: “Valine, Leucine, and Isoleucine degradation” (KEGG pathway hsa00280), “Glycolysis/Gluconeogenesis” (KEGG pathway hsa00010), “Pyruvate metabolism” (KEGG pathway hsa00620), and “Fatty acid degradation” (KEGG pathway hsa00071) (Figure 5A and Supplementary File 4). Interestingly, all these highly significant pathways that derive from proteins, carbohydrates, and lipids breakdown converge primarily in Acetyl-CoA generation, the key molecule that delivers its acetyl group for oxidation and energy production to the tricarboxylic acid (TCA) cycle (Figure 5B) and notably at least 22 of the 216 high confidence proteins (Supplementary File 2), play key enzymatic roles in the identified pathways, mostly as members of larger enzymatic complexes (marked in red in Figure 5B).

## Discussion

TAP-MS utilizing full-length WWOX as a bait under physiological conditions was employed in order to better define specific WWOX protein interactors and by extension interaction with multiprotein complexes. This work led to the identification of both well-known, but more importantly novel high confidence WWOX interactors, suggesting the involvement of WWOX in specific biological and molecular processes while delineating a comprehensive portrait of WWOX protein interactome.

We identified endoplasmic reticulum (ER), Golgi, late endosomes, protein transport, and lysosomes networks related proteins SEC23IP, SCAMP3, and VOPP1 as direct binding partners of WWOX with high interaction scores > 0.3 (Figure 2 and Table 1). SEC23IP is localized to ER exit sites (ERES) as part of the multimeric protein complex II (COPII) that coats protein transport vesicles and plays a critical function in ER to Golgi protein transport (47, 48). PROSITE analysis identified a candidate WWOX WW1 domain binding motif at ^164^PPSY^167^ residues within a larger proline-rich region (AA 135–259) of SEC23IP and importantly this same protein region was shown to be responsible for the binding to SEC23P (47). In early reports, we demonstrated that indeed WWOX predominantly localizes to the perinuclear Golgi region (3, 31). We now demonstrated that WWOX directly binds to SEC23IP *via* WW1 domain. Thus, it is possible to speculate that the WWOX-SEC23IP interaction might modulate binding of SEC23IP to the COPII complex and play an important role in ERES assembly or cargo transport.

SCAMPs are present in all post-Golgi cycling membranes and colocalize with endosomal markers thus functioning in vesicular transport. It was shown that SCAMP3 interacts with endosomal sorting complexes required for transport (ESCRT) components and plays a role in the biogenesis of multivesicular endosomes (49) and as a regulator of endosomal morphology and composition (50). SCAMP3 was suggested to be involved in negatively regulating epidermal growth factor receptor (EGFR) degradation and promoting its recycling (51). Here we showed that WWOX binds to SCAMP3 *via* WW1 domain, likely binding one of two candidate SCAMP3 WW1 binding motifs at residues ^50^PPAY^53^ or ^138^LPSF^141^.

VOPP1 previously also known as ECOP (EGFR-co-amplified overexpressed protein) was found amplified and overexpressed in multiple malignancies and thus considered to be pro-oncogenic (52, 53). VOPP1 protein structure includes a transmembrane domain and it was reported to partially co-localize with perinuclear lysosomes, suggesting that VOPP1 containing vesicles enter and participate in final lysosomal pathways (54). Very recently, Bonin et al. (46). using a yeast-two-hybrid system and co-IP reported that VOPP1 physically interacts with WWOX. They further described that upon binding, WWOX translocates to the VOPP1-containing lysosomal compartment and proposed that VOPP1 behaves as a negative regulator of WWOX tumor suppressor activity *via* this protein-protein sequestration mechanism (46). Independently, our TAP-MS studies confirmed the observations of Bonin and colleagues and validated as well the interaction by means of reciprocal co-IP assays. Of the three candidate VOPP1 PY motifs found at residues ^116^PPYY^119^, ^154^PPAY^157^ and ^162^PPPY^165^ it was concluded that the latter one was the most relevant for the interaction between these two proteins (46).

Interestingly, we have also previously shown an interaction of endogenously expressed WWOX with SIMPLE (small membrane protein of the lysosome/late endosome), which also plays a role in endosomal protein trafficking and in targeting proteins for lysosomal degradation (31). This in conjunction with the aforementioned observations involving SEC23IP, SCAMP3, and VOPP1 suggest that indeed WWOX is a prime candidate to play a relevant role in the homeostasis and regulation of perinuclear protein complexes related to protein trafficking between ERES to Golgi and from late endosomes to lysosomes.

It is noteworthy that only 16 common WWOX interactors were identified between the high throughput screen of Abu-Odeh et al. (34) and our study. Most of the identified interactors do not overlap between both datasets (Figure 4D). The causes that may account for such lack of overlap between both datasets could be due to differences in the methodological approach to identify WWOX binding partners. Abu-Odeh et al. used ectopic expression of a GST-WW1 domain fusion construct (i.e., using only a small portion of WWOX) followed by GST-pulldown and MS, while we used a full-length WWOX protein construct for our TAP-MS approach. The latter approach likely assures proper protein folding and very importantly counts with the presence of the active SDR domain which can be highly significant for proper WWOX intracellular localization (5, 35).

We believe that since TAP-MS was performed under *in-cellulo* physiological conditions, much relevant information can be gained from analyzing the data *in toto* as a portrait for identifying protein networks, pathways and cellular compartments in which WWOX likely plays a functional role. Indeed, when performing pathway analyses of WWOX interactors we identified a significant enrichment of metabolic pathways associated with proteins, carbohydrates and lipids breakdown. Thus, suggesting that WWOX likely participates in glycolysis, fatty acid degradation and other pathways that converge primarily in Acetyl-CoA generation

It is particularly intriguing that practically all members of the Pyruvate Dehydrogenase Complex (PDC), in charge of the enzymatic steps that catalyze the conversion of pyruvate (generated from glycolysis) into acetyl-CoA, can be found among the 216 proteins (Supplementary File 2), these include the PDHA1 and PDHB subunits of pyruvate dehydrogenase (i.e., PDC component E1), DLAT (dihydrolipoamide acetyltransferase, i.e., PDC component E2), and DLD (dihydrolipoamide dehydrogenase, i.e., PDC component E3). Furthermore, PDHA2 the only missing component of PDC E1, although it did not make the list of 216 proteins it was detected in replicate-1 of the TAP-MS analysis (Supplementary File 1). Additionally, PDK3 (pyruvate dehydrogenase kinase 3, not shown in Figure 5B) can also be found within our dataset (Supplementary File 2).

*Wwox KO* mice display abnormalities associated to metabolic processes including severe hypoglycemia, metabolic acidosis and additional metabolic abnormalities that lead to early death at 3–4 weeks of age, however although informative, these mouse models have been of limited used to fully understand the normal biological role of WWOX (16, 55). Importantly, evidence suggesting a direct link between WWOX loss of function and alterations in cellular respiration has been argued (7, 56). Strikingly similar to our TAP-MS findings, O'Keefe et al. (57) by using *D. melanogaster* (DMel) *Wwox* mutant models, observed that a significant number of Wwox interactors identified by a limited proteomic screen are either directly or indirectly related to metabolic pathways that precisely converge in the TCA cycle as in our findings (Figure 5B). These include many enzymes associated with glucose metabolism, lipid metabolism, ethanol metabolism, and oxidation/reduction, thus suggesting a contributing function of Wwox associated with the maintenance of aerobic metabolism (57). Furthermore, from analyzing the candidate Wwox protein interactors reported by O'Keefe et al. several orthologs and potential orthologs can be identified that closely match several of the targets identified by TAP-MS in our study. Of particular relevance is the finding of interactor DMel protein CG7430 which is the ortholog of human DLD (dihydrolipoamide dehydrogenase) precisely the E3 component of the Pyruvate Dehydrogenase Complex found in our dataset and discussed in a previous paragraph. The Drosophila proteomic screen also identified Wwox interactor CG7470 (57) ortholog of human ALDH18A1 (aldehyde dehydrogenase 18 family member A1) also found in our dataset. Various other aldehyde dehydrogenases can be found in both datasets, enzymes of much relevance in pyruvate metabolism, alcohol metabolism, fatty acids degradation and amino acids degradation (Figure 5B). Examples include Drosophila Wwox interactors DMel CG3752 and CG31075 with significant similarity to ALDH1B1 a member of enzymatic complex IUBMB/KEGG EC 1.2.1.3 also known as aldehyde dehydrogenase (NAD). Two other members of EC 1.2.13, ALDH3A2 and ADH7A1 are also found in our dataset (Supplementary File 2 and Figure 5B). This enzymatic complex plays roles in all the metabolic pathways described and shown in Figure 5B and in multiple other metabolic reactions. In conjunction, studies in human cells and Drosophila indicate that WWOX appears to play critical roles in glucose, lipid metabolism and other pathways that converge primarily in acetyl-CoA generation, which is not only the entry point to the TCA cycle for energy production, but also the key building block for the *de novo* synthesis of lipids and amino acids.

In agreement with the suggestion of WWOX playing a role or modulating cellular respiration, Abu-Remaileh and Aqeilan reported that *Wwox KO* mouse embryo fibroblasts displayed increase glucose uptake, enhanced glycolysis and reduced mitochondrial respiration resembling a “Warburg effect” like condition. It was also found that WWOX deficiency correlates with enhanced levels and activity of HIF1α over specific transcriptional targets related to glycolysis and that WWOX physically interacts with HIF1α. Based on these observations it was proposed that WWOX, *via* modulating HIF1α availability, might regulate glucose metabolism and that WWOX loss leads to activation of anaerobic glycolysis (Warburg effect) (22, 56). However, the exact mechanisms on how WWOX could affect HIF1α activity and in turn modulate cellular respiration are not clear. The association of WWOX and the described metabolic pathways also appear consistent with studies that have shown a link between WWOX and metabolic-syndrome related human traits (5, 10, 58). We also observed that gene expression profiles on samples from liver-specific *Wwox* conditional KO mice displayed significantly altered lipid metabolic profiles and increased plasma triglyceride levels, suggesting a significant role for WWOX in modulating lipid metabolism (10).

In summary, our results provide a significant lead on subsets of protein partners and enzymatic complexes with which WWOX might interact with in order to carry out its metabolic functions and other significant biological roles.

## Author Contributions

CA and JC designed research, TH, CA, and MA analyzed the data, JL performed experiments, TH and CA wrote the paper.

### Conflict of Interest Statement

The authors declare that the research was conducted in the absence of any commercial or financial relationships that could be construed as a potential conflict of interest.

## Supplementary Material

Supplementary File 1 List of WWOX interacting peptides identified by TAP-MS in replicate-1 and replicate-2.

Supplementary File 2 List of 216 WWOX protein interactors shortlisted based on MUSE and CRAPome analyses.

Supplementary File 3 List of WWOX protein interactors containing PPX(Y/F) and LPX(Y/F) motifs and motif mapping within each protein target.

Supplementary File 4 List of most significantly enriched biological pathways with list of participating WWOX interacting proteins and corresponding *p*-values.
